# Prevalence of primary biliary cirrhosis in adults referring hospital for annual health check-up in Southern China

**DOI:** 10.1186/1471-230X-10-100

**Published:** 2010-09-03

**Authors:** Haiying Liu, Yunfeng Liu, Luxia Wang, Dexing Xu, Bingliang Lin, Renqian Zhong, Sitang Gong, Mauro Podda, Pietro Invernizzi

**Affiliations:** 1 Clinical Laboratory, Guangzhou Women and Children's Medical Center, 9 Jinsui Road, Guangzhou 510623, China; 2 Clinical Laboratory, Liuhuaqiao Hospital, 111 Liuhua Road, Guangzhou 510010, China; 3 Department of Infectious Diseases, the Third Affiliated Hospital of Sun Yat-sen University, 600 Tianhe Road, Guangzhou 510630, China; 4 Laboratory Diagnostics, Changzheng Hospital, Second Military Medical University, and Clinical Immunology Center of PLA, 415 Fengyang Road, Shanghai 200003, China; 5 Division of Internal Medicine and Hepatobiliary Immunopathology Unit, IRCCS Istituto Clinico Humanitas, via A. Manzoni 113, 20089 Rozzano, Milan, Italy; 6 Department of Translational Medicine, Università degli Studi di Milano, Rozzano, Italy; 7 Division of Rheumatology, Allergy, and Clinical Immunology, University of California at Davis, 451 Health Sciences Drive, Suite 6510, Davis, CA, USA

## Abstract

**Background:**

Primary biliary cirrhosis (PBC) is an autoimmune liver disease characterized by the presence of anti-mitocondrial autoantibodies (AMA) which has an essential role also for diagnosis. In addition, also some anti-nuclear antibodies (ANA) have been shown to be highly specific PBC. The purpose of this study was to assess the prevalence of PBC among the adults referring hospital for annual health check-up in Southern China by screening sera for PBC-specific autoantibodies.

**Methods:**

AMA and ANA were screened in 8,126 adults (mean age 44 ± 15 years, 48% females) by indirect immunofluorenscence (IIF). Positive sera were tested by ELISA/immunoblotting for AMA-M2, anti-sp100 and anti-gp210. A diagnosis of PBC was re-assessed six months after the initial testing.

**Results:**

Out of 8,126 individuals 35 were positive for AMA and 79 positive for ANA. Nineteen, 4, and 3 of the subjects positive for AMA and/or ANA showed reactivity for AMA-M2, anti-sp100 or gp210, respectively, further tested with ELISA/immunoblotting. Fourteen in the 39 individuals positive for AMA at IIF, AMA-M2, anti-gp210, or anti-sp100 had abnormal cholestatic liver functional indices. One definite and 3 probable PBC diagnosis could be made in 4 cases including 3 females and 1 male after half a year.

**Conclusions:**

We found a point prevalence rate of PBC among Southern Chinese adults attending for yearly health check-up of 492 cases per million (95% CI, 128 to 1,093) and 1,558 cases per million (95% CI, 294 to 3,815) for women over 40, a finding similar to prevalence reported in other geographical areas.

## Background

Primary biliary cirrhosis (PBC) is an autoimmune liver disease leading to progressive destruction of small intrahepatic bile ducts, development of cirrhosis, and liver failure [1]. PBC affects predominantly women over middle-age [2]. It has been reported that PBC is more prevalent in some geographic areas such as Northern Europe and Northern America but much less common in Eastern Asia, Africa, and Australia [3-6]. However, it is worth noting that a rising frequency in many areas may be due to a more widespread awareness of this disease among physicians. Case series of Chinese patients with PBC have been reported from Taiwan, Hong Kong, Singapore and, more recently, also from Mainland China [6-11], with a number of Chinese patients with PBC apparently to be increasing in recent years. However, no epidemiological data have accompanied these reports.

PBC is serologically characterized by the presence of anti-mitochondrial antibodies (AMA), which are reactive mainly with E2 subunits of mitochondrial multi-enzyme complexes, the 2-oxo-acid dehydrogenase complexes comprising pyruvate dehydrogenase complex (PDC), branched chain 2-oxo-acid dehydrogenase complex (BCOADC) and 2-oxo-glutarate dehydrogenase complex (OGDC) [12]. These specific AMA are called AMA-M2 and are detectable in up to 95% of the patients [13,14]. In addition to AMA, two distinct anti-nuclear antibodies (ANA) patterns are detectable by indirect immunofluorescence (IIF), the multiple nuclear dot (MND) and rim-like (RL) patterns, for which the reactants are sp100 and the glycoprotein gp210 respectively [15,16].

The aim of this study was to investigate the prevalence of PBC among adults referring for annual health check-up by screening PBC-specific autoantibodies.

## Methods

### Subjects

A total of 8,126 citizens of Guangzhou, Southern China, aged from 18 to 83 years, with a median age of 44 ± 15 years, were consecutively enrolled in the study. 4,248 (52%) were males (median age of 46 ± 15 years), 3,878 (48%) were females (median age 41 ± 14 years), and 1,926 (24%) were aged over 40 years. They underwent a yearly health check-up at the Liuhuaqiao Hospital from June to September 2006, including a physical examination, routine blood tests, abdominal ultrasonography, chest X ray, electrocardiogram, and serum markers of hepatitis B (HBsAg). The male to female ratio in the current study (1.1) reflects the general sex distribution in Guangzhou (1.0) investigated at the end of 2005 http://www.civic-exchange.org/eng/upload/files/200806_AirQualityPublicHealth.pdf.

This study was in compliance with the Helsinki Declaration http://www.wma.net/e/policy/b3.htm and was approved by the regional ethical committee of the Guangdong province, China. All subjects signed an informed consent to be enrolled and all data were dealt with in an anonymous way.

### Screening of AMA and ANA

**Figure 1:**
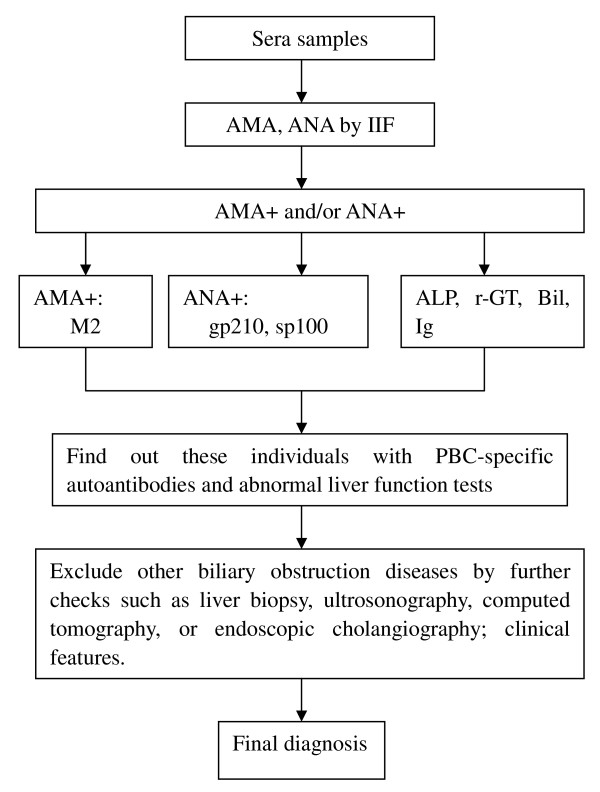
**Experimental procedure of screening autoantibodies and diagnosis**.

Figure 1 shows the experimental procedure of the study. Pools of sera from 6 subjects were prepared from the 8,126 available sera, and each pool was tested for AMA and ANA reactivity by IIF using HEp-2 cells as substrate according to the instructions of the manufacturer (Euroimmun, Luebeck, Germany). HEp-2 cell lines were used for screening of both ANA and AMA although the preferable substrate is that of combination of rodent tissues [17,18]. Sera from each positive pool were then individually tested with the same approach in order to identify each individual positive serum sample. A coarse speckled cytoplasmic staining of mitochondria HEp-2 cells was read as AMA positive, but AMA was then further confirmed by IIF on rat kidney tissues (Euroimmun).

### Detection of AMA-M2, anti-gp210 and anti-sp100

Sera positive for AMA and/or ANA by IIF were further tested for AMA-M2, anti-gp210 and anti-sp100. AMA-M2 in serum samples diluted 1:100 were determined using ELISA kits with wells coated with recombinant fusion protein containing the immunodominant epitopes of human PDC-E2, BCOADC-E2 and OGDC-E2 (Fuchunzhongnan Biotech, Shanghai, China). Anti-sp100 and anti-gp210 were detected at a dilution of 1:100 by an immunoblotting assay with recombinant sp100 and gp210 proteins (IMTEC, Berlin, Germany).

### Laboratory indices

The routine diagnostic laboratory indices included liver function such as serum total protein, albumin, alanine aminotransferase (ALT), aspartate aminotransferase (AST), total cholesterol, triglyceride, and immunoassays for hepatitis B virus (HBV) infection (HBsAg, anti-HBs, HBeAg, anti-HBe and anti-HBc). Sera positive for PBC-specific autoantibodies were additionally tested for antibodies against other hepatitis viruses (HAV, HCV and HEV), serum immunoglobulins (Ig), total bilirubin (TBil), alkaline phosphatase (AP), and gamma-glutamyl transpeptidase (γ-GT).

### Diagnosis of PBC

All subjects positive for at least one of the PBC-specific antibodies and with abnormal results for liver function tests were advised to visit hepatologists for further checks, and the laboratory indices were retested after six months. A diagnosis of PBC was made according to criteria recommended by the European Association for the study of the Liver (EASL) [19]: unexplained elevation of ALP and presence of AMA (≥ 1:40) and/or AMA-M2.

### Statistical analysis

SPSS13.0 software was applied to calculate 95% confidence intervals (CI) for frequencies of autoantibodies and the point prevalence rate of PBC. χ^2 ^test was used to compare categorical variables. Two-tailed *P *values of less than 0.05 were considered to be statistically significant.

## Results

### AMA and ANA frequencies

Out of the 8,126 subjects, 104 were tested positive for AMA and/or ANA at IIF. Both ANA and AMA were tested by HEp-2 cell lines for financial constraints and because this approach allowed to obtain information on ANA patterns. Among them, 35 (0.43%, 95% CI: 0.30%-0.58%) were positive for AMA, 15 males and 20 females, mean age, 55 ± 15 (range, 23-83 years); 79 (0.97%) tested ANA positive and the frequencies of different ANA patterns were shown in table 1. A speckled pattern was the most common (44.3%); MND and RL patterns were observed in 11 (13.9%) and 4 subjects (5.1%), respectively. Sera from 10 ANA-positive individuals were concurrently AMA positive.

**Table 1:** Indirect immunofluorescence patterns of 79 subjects with anti-nuclear antibodies (ANA) on HEp-2 cells.

ANA Pattern	**Positive no**.	%
Speckled	35	44.3
Nucleoli	13	16.5
Centromere	12	15.2
Multiple nuclear dots	11	13.9
Homogeneous	7	8.9
Rim-like	4	5.1

### AMA-M2, anti-sp100 and anti-gp210 frequencies

**Figure 2:**
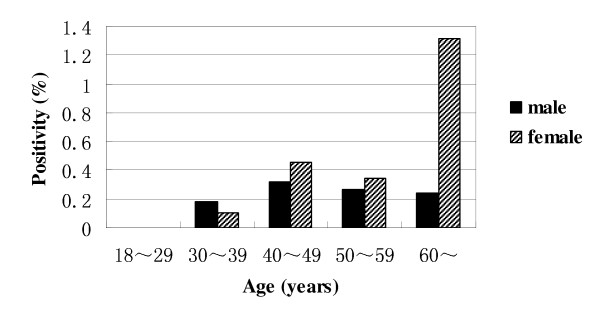
**Distribution of PBC-specific autoantibodies in male and female at different ages**. None of PBC-specific autoantibodies (AMA-M2, anti-sp100 or anti-gp210) was detected in the individuals at the age of 18~29. There was no significant difference in the frequency of PBC-specific autoantibodies between male and female at all ages (*P *> 0.05).

Twenty-two of the 104 sera (22%) that tested positive for AMA and/or ANA by IIF contained autoantibodies directed against specific mitochondrial and/or nuclear antigens as shown in table 2. In particular, 19, 4, and 3 of these 104 sera were found to be positive for anti-AMA-M2, anti-sp100 and anti-gp210, respectively. Distribution of PBC-specific autoantibodies including AMA-M2, anti-sp100 and anti-gp210 in male vs. female at different ages was shown in figure 2. None of specific autoantibodies (anti-AMA-M2, anti-sp100 or anti-gp210) was detected in the individuals at the age of 18~29. These autoantibodies occurred seldom in adults before age of 40 and the positive rates did not increased with age (*P *> 0.05). Although the PBC-specific autoantibodies prevalence in female over age of 40 was 0.62% and 1.32% for women over 60, several folds of that found among males of similar age, there was no statistical difference between female and male at all ages (*P *> 0.05).

**Table 2:** Indirect immunofluorescence (IIF) patterns according to specific immunoreactivities (AMA-M2, anti-sp100 and anti-gp210) in the 104 sera positive for AMA and/or ANA.

IIF patterns	n	M2	sp100	gp210	M2+sp100	M2+sp100+gp210
MND	3	1	1	0	1	0
Rim-like	1	0	0	1	0	0
AMA	15	12	0	1	1	1
AMA+MND	2	2	0	0	0	0
AMA+Speckled	1	1	0	0	0	0
Total	22	16	1	2	2	1

*Note*. Abbreviations are same as that in the text.

### Clinical characteristics of subjects with PBC-specific antibodies

There was 17 individuals AMA positive at IIF in whom none of M2, anti-sp100 or anti-gp210 was detected. Overall, 39 of the 8,126 sera (0.48%, 95% CI: 0.34%-0.64%) were tested positive for AMA at IIF, AMA-M2, anti-gp210, or anti-sp100, that is all the known PBC-specific serum immunoreactivities [2,15]. Fourteen of these 39 individuals (8 of 14 were females) had at least one abnormal test of liver function. None showed signs of current infection with a hepatitis virus. All 14 subjects with abnormal tests of liver function were contacted for further clinical evaluation, but only one, a 60 year old woman with fatigue and elevated serum immunoglobulins, total bilirubin, AP, and γ-GT, underwent ERCP and liver biopsy. ERCP did not disclose biliary tract abnormalities, and liver biopsy showed periportal hepatitis and bile duct proliferation indicative of a histological stage II PBC [20]. Another three cases without any related clinical signs, 2 females and one male aged 41, 64, 47 respectively, maintained abnormal liver functional indices after six months and also fulfilled the diagnostic criteria of PBC proposed by EASL.

## Discussion and conclusions

The first patient to be recorded with PBC in Mainland China was reported in 1958 and only an additional 48 cases were later on reported from 1980 to 2000 [21]. Although there have been an increasing number of reported diagnoses, PBC is still thought to be very rare in Asian populations. Moreover, there is a lack of solid epidemiological information relevant to this issue [3]. Therefore, we took advantage of annual health check-up data and designed a study to estimate the prevalence of PBC in the adult population of a defined area of Southern China by screening for all the known PBC-specific serum autoantibodies [15].

Four individuals were diagnosed as PBC, 3 females and 1 male, among 8,126 adults (over age 18) resident in the Southern Chinese city of Guangzhou who attended for an annual health check-up; the rate of hepatitis B virus in this population was 8.1%. The point prevalence rate of PBC was 492 cases per million (95% CI: 128 to 1,093), among the highest reported in other geographical regions [5,22-26]. By considering the risk of PBC for women over 40 years, i.e. a "high risk group", a prevalence of 1,558 cases per million (95% CI: 294 to 3,815) was observed.

Unfortunately, we could not confirm the diagnosis of PBC by means of liver biopsy in all the subjects with cholestasis and the presence of PBC-specific autoantibodies. But the serum autoantibodies we determined are highly specific for PBC [15], which allowed us to screen adults in a relatively natural distribution of the general population, thus providing a fair estimate of the real prevalence of PBC in Mainland China.

The prevalence of PBC found in our study is similar to that reported in an early evaluation from Italy, in which diagnosis was based on ultrasound scanning, elevated AP and presence of AMA [27], but much lower than the estimated prevalence of PBC for middle-aged women in Japan [28]. However, it is to note that in this latter study the screening comprised only increase in serum γ-GT levels among 4,048 asymptomatic women attending for an annual health check-up. It is evident that prevalence obtained from laboratory parameters are higher than prevalence derived from studies using a physician survey and review of hospital records [29-33]. The reason may be that laboratory methods can identify asymptomatic cases that would be overlooked by case-finding; for instance, the four PBC patients identified in our study including the patient who already developed symptoms had never encountered a hepatologist. Overall, the variation in PBC prevalence among different studies may be attributable to differences in study populations and periods and the methods of case ascertainment [5].

Jiang and colleagues identified 3 (0.06%) PBC cases from 5,011 adults from eastern China [34]. Lately, 38 PBC cases were reported in Singapore, a small Asian city-state with population of 4 million and ethnic distribution of 76% Chinese [11]. Based on the current and early findings, the prevalence of PBC in Asia, especially in Mainland China, is not as rare as might be expected, being comparable with high prevalence sites in Northern Europe and the United States of America [5].

In areas like Mainland China where there is still lack of registry system for healthcare and PBC is not well recognized by clinical doctors the need of data on disease prevalence and incidence is crucial. We are now reporting data from a screening of PBC starting from combination of PBC specific autoantibodies in adults at random with age and sex in a relatively natural distribution. We are aware that our cross-sectional observational study has a number of limitations, also because allowed us to identify only a small absolute number of cases. Theoretically, a population-based study is the best study design to define the prevalence of a disease, and efforts have to be done to accomplish such studies in the near future.

Intriguingly, the current study confirms the observation of a nearly equal male to female ratio found in large-scale screening studies, as we have recently highlighted [35]. On the contrary, studies based on case-finding methods reported a strong female preponderance, possibly because they account only for already diagnosed cases [35]. Although the reasons for this discrepancies remain unknown, clearly rely to the different study designed used.

In summary, our data suggest that PBC is not as rare as expected before with the point prevalence rate of PBC in Chinese which is quite comparable to that reported in other geographical areas.

## Competing interests

The authors declare that they have no competing interests.

## Authors' contributions

HL and PI ideated the study. HL, YL, LW, DX, and BL designed the study and analyzed the data. HL, RZ, SG, MP, and PI were responsible for writing the manuscript and revising it critically for important intellectual content. All authors read and approved the final manuscript.

## Pre-publication history

The pre-publication history for this paper can be accessed here:

http://www.biomedcentral.com/1471-230X/10/100/prepub
